# Comprehensive Analysis of Metabolic Genes in Breast Cancer Based on Multi-Omics Data

**DOI:** 10.3389/pore.2021.1609789

**Published:** 2021-08-02

**Authors:** Yu Hua, Lihong Gao, Xiaobo Li

**Affiliations:** Department of Nursing, The First Affiliated Hospital of China Medical University, Shenyang, China

**Keywords:** breast cancer, prognosis, mutation, metabolism, WGCNA

## Abstract

**Background:** Reprogramming of cell metabolism is one of the most important hallmarks of breast cancer. This study aimed to comprehensively analyze metabolic genes in the initiation, progression, and prognosis of breast cancer.

**Materials and Methods:** Data from The Cancer Genome Atlas (TCGA) in breast cancer were downloaded including RNA-seq, copy number variation, mutation, and DNA methylation. A gene co-expression network was constructed by the weighted correlation network analysis (WGCNA) package in R. Association of metabolic genes with tumor-related immune cells and clinical parameters were also investigated.

**Results:** We summarized 3,620 metabolic genes and observed mutations in 2,964 genes, of which the most frequently mutated were PIK3CA (51%), TNN (26%), and KMT2C (15%). Four genes (AKT1, ERBB2, KMT2C, and USP34) were associated with survival of breast cancer. Significant association was detected in the tumor mutation burden (TMB) of metabolic genes with T stage (*p* = 0.045) and N stage (*p* = 0.004). Copy number variations were significantly associated with recurrence and prognosis of breast cancer. The co-expression network for differentially expressed metabolic genes by WGCNA suggested that the modules were associated with glycerophospholipid, arachidonic acid, carbon, glycolysis/gluconeogenesis, and pyrimidine/purine metabolism. Glycerophospholipid metabolism correlated with most of the immune cells, while arachidonic acid metabolism demonstrated a significant correlation with endothelial cells. Methylation and miRNA jointly regulated 14 metabolic genes while mutation and methylation jointly regulated PIK3R1.

**Conclusion:** Based on multi-omics data of somatic mutation, copy number variation, mRNA expression, miRNA expression, and DNA methylation, we identified a series of differentially expressed metabolic genes. Metabolic genes are associated with tumor-related immune cells and clinical parameters, which might be therapy targets in future clinical application.

## Introduction

Breast cancer is the most frequently diagnosed cancer in women and one of the leading cause of cancer-related death all around the world [1, 2]. Early diagnosed breast cancer is curable in most patients with no metastatic disease [3, 4]. However, advanced breast cancer with metastatic status is still regarded as incurable although multiple therapies are available [5, 6]. On the basis of the clinical subtype of breast cancer, current therapies include endocrine therapy, anti-HER2 targeting as well as chemotherapy [7–9]. Various aspects including genetic mutations, epigenetic alternations, and environmental factors have been reported to be implicated in the development of breast cancer [10, 11].

Reprogramming of cell metabolism is one of the most important hallmarks of multiple cancer types including breast cancer [12, 13]. Increasing studies highlighted the many different metabolic choices between cancer cells and normal cells [14, 15]. Breast cancer cells sustain the transformed status and survive the specific tumor microenvironment by remodeling the metabolism network [16, 17]. Aberrant metabolic status further influences cellular signaling pathways and the environment to facilitate breast cancer progression [18]. The specific metabolic characteristics offer possible targets for breast cancer of metabolic pathways and biomarkers for future diagnosis and prognosis in clinical investigations.

Considering the critical role of metabolism in the initiation and progression of breast cancer, an increasing number of studies focused on the close implication of metabolism in different aspects of breast cancer. Interaction between the estrogen pathway and core metabolic regulators changes the metabolism of breast cancer cells and facilitates cancer cells to adapt to nutrient insufficiency and high acidity [13]. The regulation of JAK/STAT3 in fatty acid β-oxidation contributes to breast cancer cell stemness and chemoresistance [19]. In addition, CD44ICD (CD44 molecule (Indian blood group)) leads to breast cancer stemness through the PFKFB4(6-phosphofructo-2-kinase/fructose-2,6-biphosphatase 4)-mediated metabolism of glucose [20]. Metabolic enzyme PFKFB4 promotes transcriptional co-activator SRC3 (nuclear receptor coactivator 3) for breast cancer carcinogenesis and is associated with poor prognosis of breast cancer [21].

In order to elucidate the alternations of metabolic genes in the development and prognosis of breast cancer, we performed comprehensive analysis of metabolic genes in breast cancer based on multi-omics data. Somatic mutation, copy number variation, mRNA expression, miRNA expression, and DNA methylation data were downloaded from The Cancer Genome Atlas (TCGA) and systematically analyzed. Weighted correlation network analysis (WGCNA) was used to construct a co-expression network for these differentially expressed metabolic genes. In addition, association of metabolic genes with tumor-related immune cells and clinical parameters were also investigated to further reveal the potential application of targeting metabolic genes in clinical applications.

## Materials and Methods

### Data Collection

Metabolic genes were obtained from Fluxer (https://fluxer.umbc.edu/). The RNA sequencing, copy number variations, mutation, methylation, miRNA expression, and clinical data of breast invasive carcinoma (BRCA) patients in TCGA datasets were downloaded from UCSC XENA (https://xena.ucsc.edu/). The level of gene expression was measured as transcripts per million reads (TPM). Clinical data included TNM stage, cancer stage, recurrence event, and survival information.

### Somatic Mutation and Copy Number Analysis

Since gene mutations do not necessarily affect gene function, we selected loss of function (LOF) mutations for the following analysis in order to illustrate the importance of mutations. Among the mutation types, we defined FrameShiftIns, FrameShiftDel, NonsenseMutation, NonstopMutation, SpliceSite, and TanslationStart_Site to describe mutations that were LOF. First, we selected mutant genes whose mutation samples were greater than 20 for prognostic analysis. We observed whether gene mutations influenced the prognosis of TCGA-BRCA using Kaplan-Meier log rank tests. Furthermore, we used cometExactTest, an R package, to analyze whether the mutations of prognostic-related genes were mutually exclusive. In order to further understand the impact of metabolic gene mutations on clinical characteristics, we used the above gene mutation information to calculate the tumor mutation burden (TMB) of each sample. Then, the tumor mutation burden and clinical information was evaluated. Furthermore, we evaluated the relationship between the metabolism-related fraction genome altered and clinical parameters.

### RNA-Seq Analysis

For the tumor-normal comparison, we performed a differential expression analysis using the Deseq2 package and defined differential expression genes using FDR <0.05 and |logFC| > 1. In order to understand the impact of metabolism-related genes on breast cancer, we selected metabolic-related genes with different expressions for weighted gene co-expression network (WGCNA) analysis. The gene co-expression network was constructed by the WGCNA package in R. Power values were screened out by the WGCNA algorithm in the construction of co-expression modules. Scale independence and average connectivity analysis of modules with different power values were performed by a gradient test (power value ranging from 1 to 20). An appropriate power value was determined when the scale independence value was equal to 0.9. The WGCNA algorithm was then used to construct the co-expression network and extract the gene information in the most relevant module. After clustering gene expression analysis, we first determined the specific metabolic pathways of each module through pathway enrichment analysis. Furthermore, we evaluated the correlation between each module and immune cells, which were calculated through the MCPcounter and GSVA algorithm. Finally, we analyzed the correlation between related modules and clinical features and whether it had an impact on the prognosis.

### miRNA Expression Analysis

We obtained normalized miRNA expression data from Genomic Data Commons. In order to study the mechanisms underlying dysregulated metabolism-related genes in breast cancer, we identified master miRNA regulators for metabolism-related genes based on two criteria. First, a miRNA had to have at least one seed region (2–8-mer) matched to the 3′UTR of any metabolism-related gene. Second, the Pearson correlation of miRNA with the expression of the target genes had to be statistically significant (*p* < 0.05 and R2 < −0.5). Cytoscape was then used to visualize the network of miRNA and metabolism-related genes.

### DNA Methylation Analysis

For methylation, the probe in the promoter region were selected. For each gene, one DNA methylation probe was selected based on the correlation with its mRNA expression level. If multiple probes for a gene were available, the probe that had the most negative correlation value was selected. We identified master methylation regulators for metabolism-related genes based on one criteria that the Pearson correlation of methylation with the expression of the target gene was statistically significant (*p* < 0.05 and *r* < −0.3).

### Statistical Analysis

We applied R language for analysis and statistics. T test was used to compare the distribution between two groups. Analysis of variance was performed for comparison among three or more groups, and Chi-square test was used to compare the distribution difference between two categories. Survival analysis was carried out through Kaplan-Meier methods and compared by the log-rank test. A two-tailed *p* value <0.05 was statistically significant.

## Results

### Genomic Changes of Metabolic Genes in Breast Cancer

**FIGURE 1:**
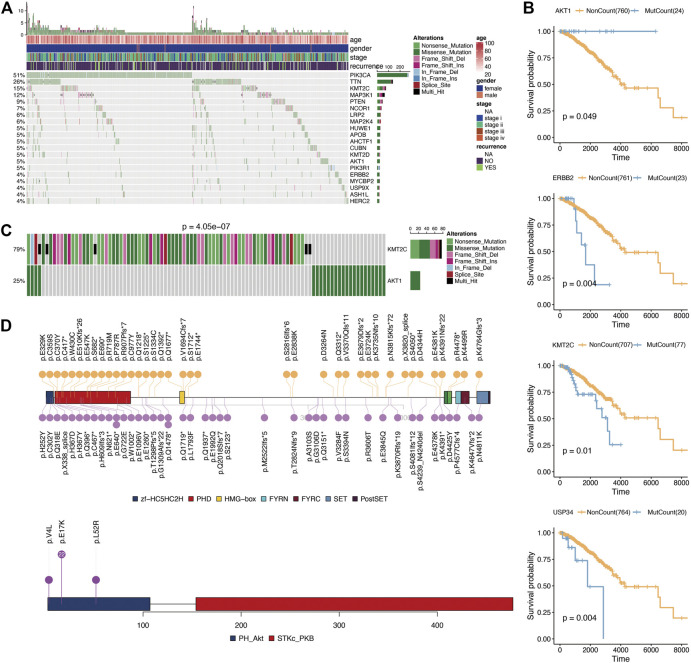
Mutation analysis of metabolism-related genes. **(A)**, Oncoplot for the top 20 mutation metabolism-related genes. **(B)**, Kaplan-Meier prognostic analysis of gene mutation or not in breast cancer. **(C)**, Mutual exclusion analysis using CometExactTest R package between AKT1 and KMT2C. **(D)**, Specific mutation positions of AKT1 and KMT2C.

According to the Fluxer database, we summarized 3,620 metabolic genes (Supplementary Table S1). We performed further analysis using these 3,620 genes as metabolism-related genes. For genomic analysis, we observed mutations in 2,964 genes. As shown in Figure 1A, the most frequently mutated metabolic genes were phosphatidylinositol-4,5-bisphosphate 3-kinase Catalytic subunit alpha (PIK3CA) (51%), tenascin N (TNN) (26%), and lysine methyltransferase 2C (KMT2C) (15%). Next, we performed prognosis analysis for metabolic genes with mutations over 20. After prognosis analysis, we found four genes (AKT1 (AKT serine/threonine kinase 1), ERBB2 (Erb-B2 receptor tyrosine kinase 2), KMT2C (lysine methyltransferase 2C), and USP34 (ubiquitin specific peptidase 34)) associated with survival of breast cancer (Figure 1B; Supplementary Table S2). Among them, the prognostic analysis of AKT1 gene mutation was most related to breast cancer. And breast cancer patients had a longer survival time in the AKT1 mutation group. Considering the influence between different gene mutations, we then performed repulsion analysis of these prognosis-related metabolic genes. Repulsion was revealed among different genes (Supplementary Table S3), of which the most significant effect was found in the AKT1-KMT2C pair (Figure 1C). As a result, we checked the mutation information of these two genes in detail and found that AKT1 gene mutations mainly occur at p.E17K while KMT2C mutations are distributed dispersedly (Figure 1D).

### Tumor Mutation Burden (TMB) and Fraction Genome Alternation (FGA) of Metabolic Genes in Breast Cancer

In order to further elucidate the association between mutations of metabolic genes with breast cancer, we calculated the TMB using the mutation information.

**FIGURE 2:**
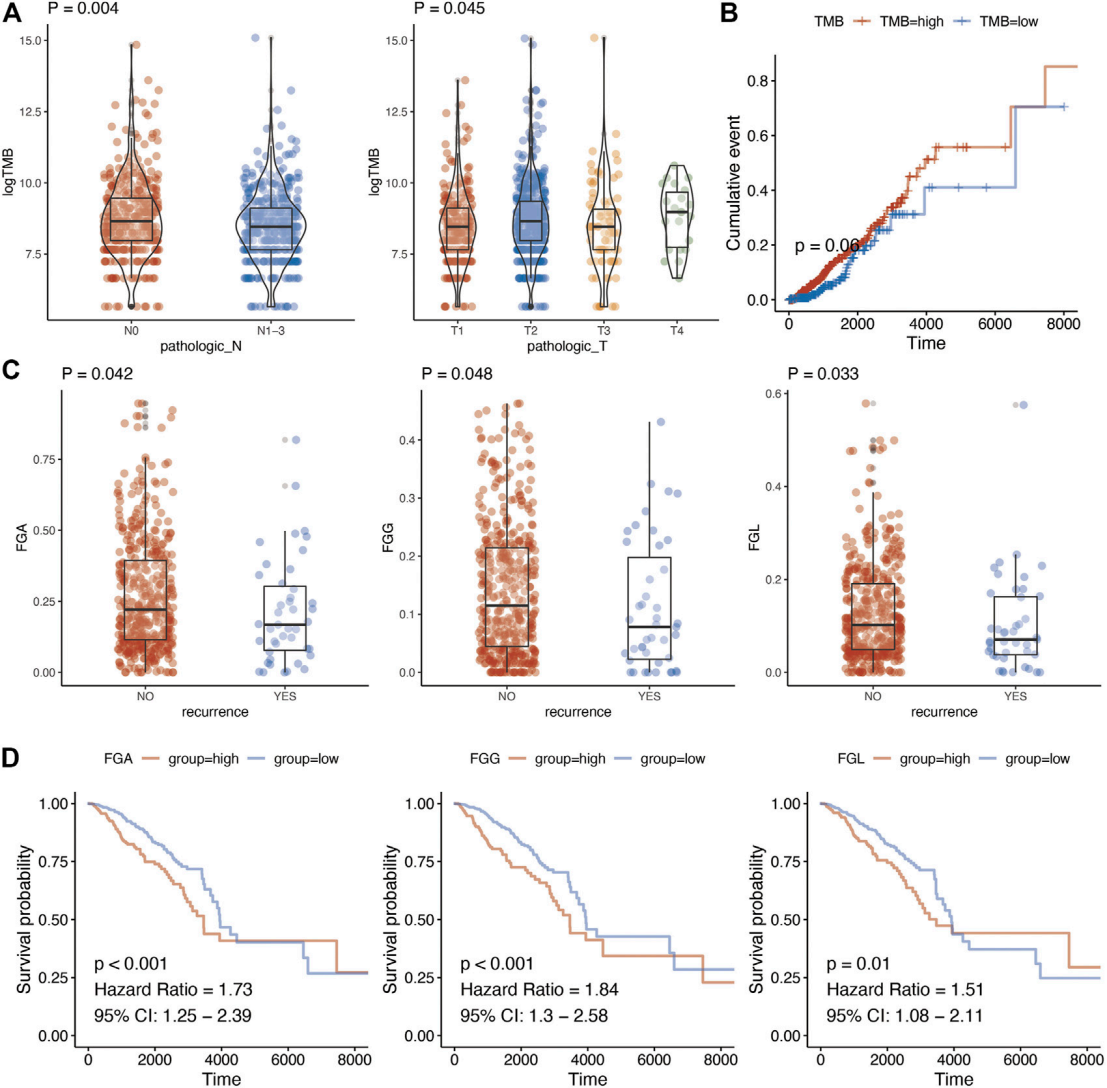
Genome characteristics and clinical parameter analysis of metabolism-related genes. **(A)**, Relationship between tumor mutation burden of metabolism-related genes and N stage and T stage. T test was used to analyze the difference of expression between two groups. **(B)**, Kaplan-Meier prognostic analysis of tumor mutation burden of metabolism-related genes. **(C)**, Changes in the copy number fragments of metabolism-related genes are associated with tumor recurrence. T test was used to analyze the difference of expression between two groups **(D)**, Changes in copy number fragments of metabolism-related genes were found to be associated with breast cancer prognosis using Kaplan-Meier methods.

The relationship of TMB with TNM stage, relapse, and prognosis was analyzed. Finally, a significant association was detected between TMB of metabolic genes with T stage (*p* = 0.045) and N stage (*p* = 0.004) (Figure 2A; Supplementary Table S4). In addition, we analyzed whether there were differences in TMB among the groups in the classic PAM50 molecular classification of breast cancer. After analysis, it is found that there were differences in metabolism-related TMB among different PAM50 groups (Supplementary Figure S1, *p* < 0.001). As for prognosis, TMB demonstrated a borderline association with breast cancer prognosis (*p* = 0.060) (Figure 2B). The genomic changes also contained copy number variations other than gene mutations. We calculated fraction genome alternation (FGA), fraction genome gain (FGG), and fraction genome loss (FGL) using copy number variation data of metabolic genes in breast cancer. This suggested that FGA, FGG, and FGL were significantly associated with recurrence of breast cancer (Figure 2C, *p* = 0.042, *p* = 0.048, and *p* = 0.033, respectively). Moreover, there were also differences in FGA/FGG/FGL among different PAM50 molecular types (Supplementary Figure S2, *p* < 0.001). In addition, high FGA (HR = 1.73, 95%CI = 1.25–2.39, *p* < 0.001), FGG (HR = 1.84, 95%CI = 1.30–2.58, *p* < 0.001), and FGL (HR = 1.51, 95%CI = 1.08–2.11, *p* = 0.010) predicted worse prognosis of breast cancer (Figure 2D; Supplementary Table S5).

### Functional Analysis of Metabolic Genes in Breast Cancer

**FIGURE 3:**
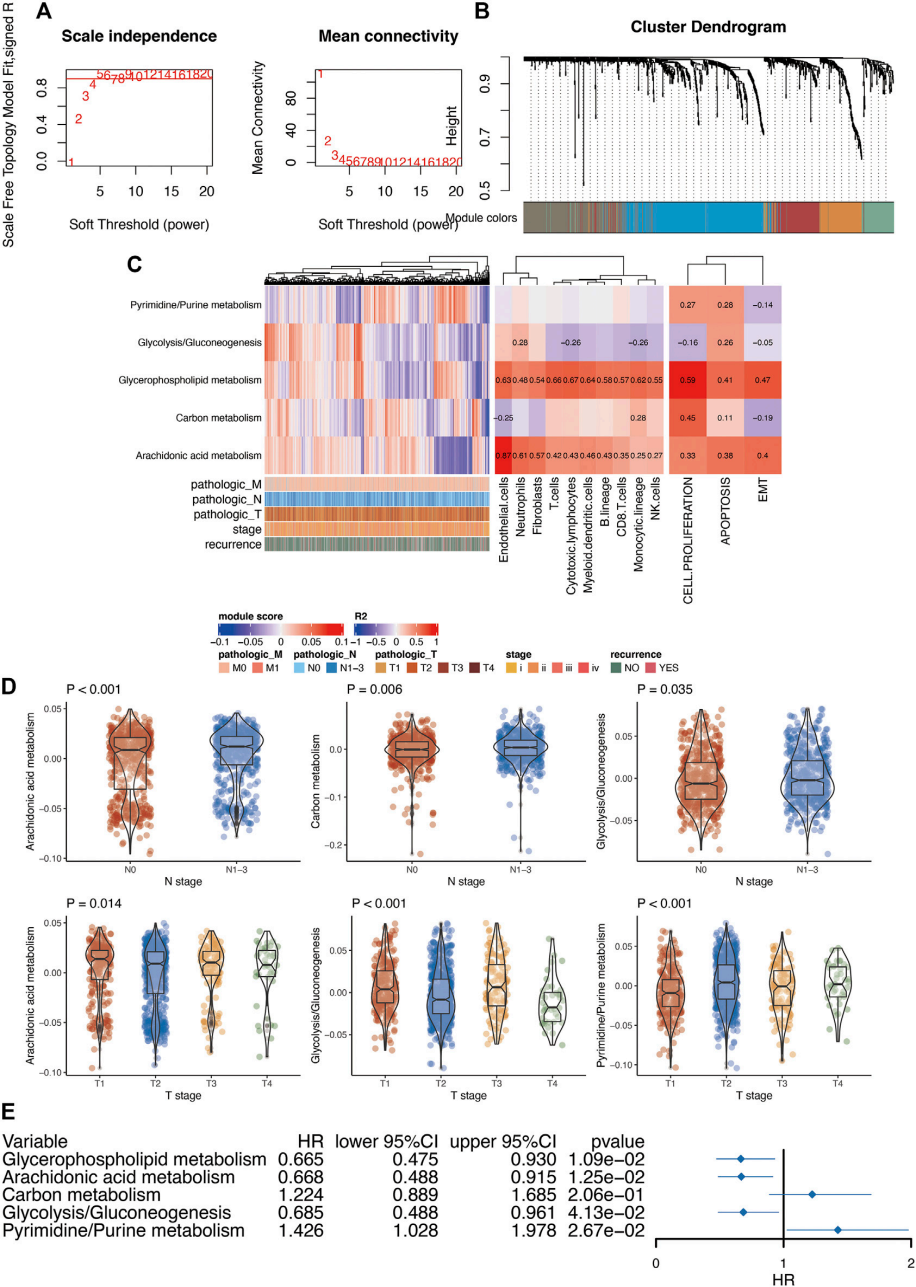
Functional analysis of metabolism-related genes in breast cancer. **(A)**, Soft threshold selection of WGCNA. **(B)**, Module clustering analyzed by WGCNA. **(C)**, The relationship between metabolic-related modules and immune infiltration and tumor phenotype. Pearson correlation analysis was used to analysis the relationship between modules and immune infiltration. **(D)**, The relationship between metabolism-related modules and clinical features of breast cancer. Prognostic analysis of metabolic-related modules. T test was used to analyze the difference of expression between two groups. ANOVA test was used to analyze the difference of expression among more than two groups. **(E)**, Kaplan-Meier prognostic analysis of the modules generated from WGCNA analysis.

First, we performed differential analysis of metabolic genes between breast cancer tissues and normal tissues. After differential expression analysis, we found that 466 genes were highly expressed in breast cancer, and 358 genes were lowly expressed in breast cancer (Supplementary Table S6). Furthermore, in order to further understand the role of these differentially expressed genes in breast cancer, we used WGCNA analysis to construct a co-expression network for these differentially expressed metabolic genes. After the preliminary data evaluation, we choose 5 as the candidate threshold for co-expression network construction (Figure 3A). Gene modules were then constructed according to the expression relationship between metabolic genes (Figure 3B). Finally, we obtained five modules after WGCNA analysis. Among these modules, gene numbers ranged from 87 to 187. Because WGCNA has a certain degree of randomness in the analysis of the modules, in order to be conservative in the standard model analysis, we used the Chin 2006 dataset to conduct a conservative analysis of the five modules obtained. After analysis [22], it was found that the above five modules were all stably expressed (Supplementary Figure S3). Through the pathway enrichment analysis of these modules, we found that these modules were associated with glycerophospholipid metabolism, arachidonic acid metabolism, carbon metabolism, glycolysis/gluconeogenesis, and pyrimidine/purine metabolism.

In order to understand the relationship between these modules and tumor-related immune cells, we first used the MCPcounter algorithm to evaluate the immune infiltration of each sample. Further analysis of the correlation between each module and immune infiltration indicated certain relationships of glycerophospholipid metabolism with most of the immune cells. In addition, arachidonic acid metabolism demonstrated a significant correlation with endothelial cells (Figure 3C). Next, we also used the GSVA algorithm to assess tumor-related phenotype scores (cell proliferation, apoptosis, and EMT (epithelial-mesenchymal transition)) in breast cancer patients. The final analysis results suggested a significant correlation between glycerophospholipid metabolism and cell proliferation.

We also analyzed the relationship between these modules and clinical parameters. N staging was mainly related to arachidonic acid, glycolysis/gluconeogenesis, and carbon metabolism. In addition, T stage was mainly related to arachidonic acid, glycolysis/gluconeogenesis, and pyrimidine/purine metabolism (Figure 3D). In addition, all metabolic pathways were related to PAM50 (Supplementary Table S7). Finally, we performed prognosis analysis of each module and found that multiple metabolic modules were related to prognosis except for carbon metabolism (Figure 3E).

### Possible Regulatory Mechanisms of Metabolic Genes

**FIGURE 4:**
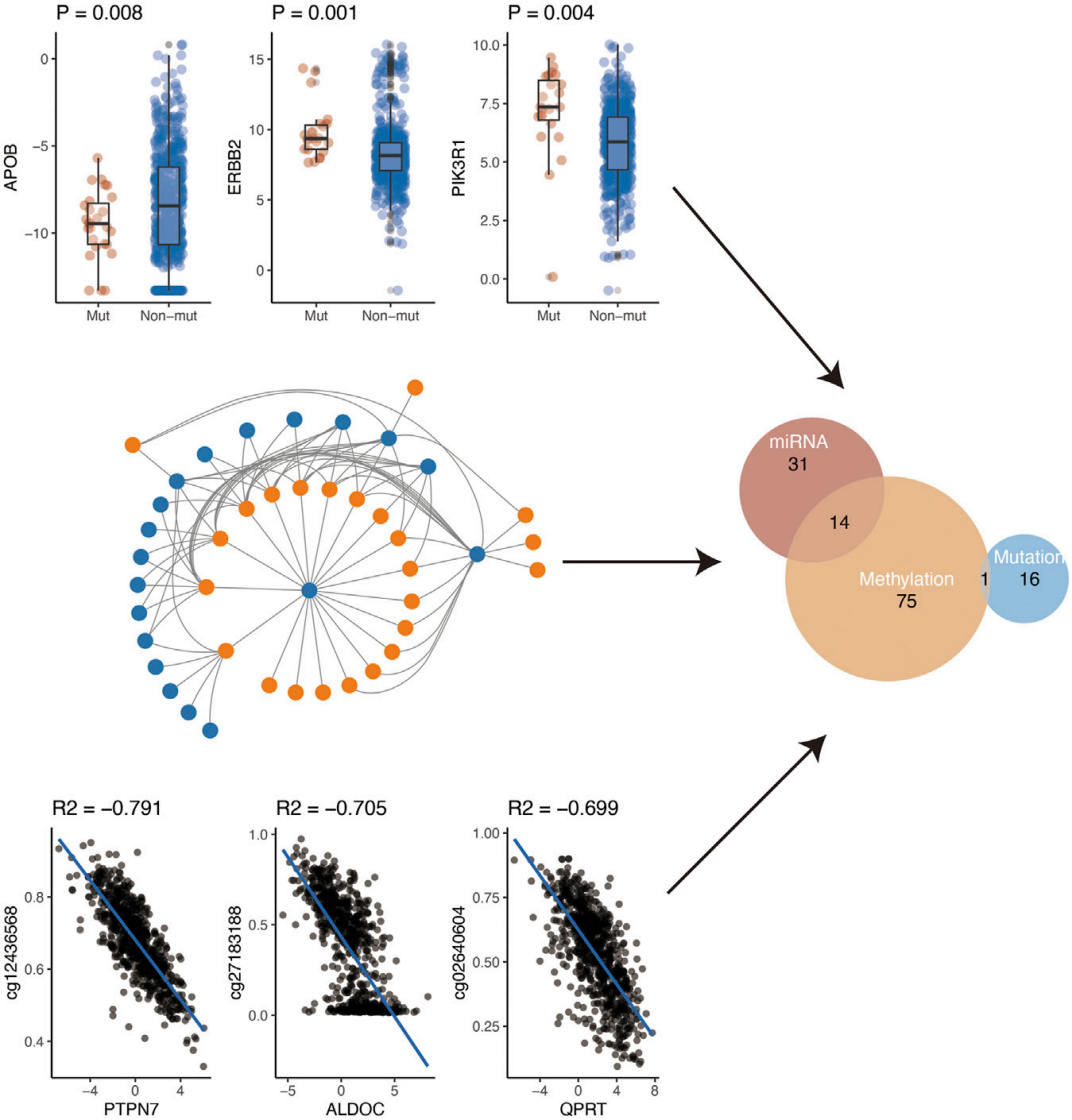
Potential regulatory mechanism of metabolism-related genes.

Gene expression is regulated by various factors including promoter methylation, miRNA, and gene mutation. Using the comprehensive data in TCGA, we further explored the possible regulatory factors (mutation, miRNA regulation, and methylation regulation) of these differential genes. First of all, we analyzed the effect of gene mutation on gene expression. We found that the expression of 17 genes was affected by their mutations. Secondly, we analyzed the negative regulation of miRNA on gene expression. After the analysis, we found that a total of 45 genes were negatively regulated by miRNA. Finally, we investigated whether methylation of the gene promoter region affected gene expression. Finally, we found that 90 genes might be regulated by their methylation. In conclusion, according to the above three analyses, a total of 137 genes were regulated by the above three mechanisms (Figure 4). Specifically, methylation and miRNA jointly regulated 14 genes while mutation and methylation jointly regulated one gene (PIK3R1) (Supplementary Table S8).

## Discussion

Abnormal metabolism of breast cancer cells remodels the tumor microenvironment leading to cancer vascularization and disturbs tumor immunity to participate in cancer development [23]. Considering the close implication of metabolic genes in the initiation, progression, and prognosis of breast cancer, we conducted a comprehensive analysis of metabolic genes of breast cancer covering somatic mutation, copy number variation, mRNA expression, miRNA expression, and DNA methylation data. The results indicated that metabolic genes were associated with multiple signaling pathways, immune cells, and prognosis of breast cancer.

For genomic analysis, we observed mutations in 2,964 genes of 3,620 metabolic genes. The most frequently mutated metabolic genes were PIK3CA (51%), TNN (26%), and KMT2C (15%). After prognosis analysis, we found four genes (AKT1, ERBB2, KMT2C, and USP34) associated with survival of breast cancer. The AKT1 mutation is very important in breast cancer. Previous studies have also found that AKT1 mutations are closely related to breast cancer [24]. In our analysis, it was found that AKT1 mutations can increase the survival time of patients. The possible reason is that an AKT1 mutation can inhibit cell survival and proliferation, and promote tumor cell apoptosis [25]. Considering the influence between different gene mutations, we then performed repulsion analysis of these prognosis-related metabolic genes. Repulsion was revealed among different genes, of which the most significant effect was found in the AKT1-KMT2C pair. It has been reported that the PI3K pathway is a commonly altered signaling pathway in breast cancer, making it a possible therapy target [26]. As for KMT2C mutation, it has been suggested that KMT2C is a core regulator of ERα activity, which modulates the dependence of estrogen in breast cancer [27]. In addition, in metabolic pathways, both ATK1 and KMT2C are related to lipid metabolism. The mutual exclusion of these two genes may have different effects on lipid metabolism.

For association between mutations of metabolic genes with breast cancer, we calculated the TMB using the mutation information. A significant association was detected between the TMB of metabolic genes with T stage (*p* = 0.045) and N stage. TMB also demonstrated a borderline association with breast cancer prognosis. As for copy number variations, we suggested that FGA, FGG, and FGL were significantly associated with recurrence of breast cancer. In addition, high FGA, FGG, and FGL predicted worse prognosis of breast cancer. A previous study has suggested TMB as a predictor for immune-related survival in breast cancer [28]. In addition, certain copy number alternations possess critical implications for designing novel therapeutic strategies [29].

For differentially expressed analysis of metabolic genes in breast cancer, we identified that 466 genes were highly expressed in breast cancer, and 358 genes were lowly expressed in breast cancer. We used WGCNA analysis to construct a co-expression network for these differentially expressed metabolic genes. Gene modules were then constructed according to the expression relationship between metabolic genes. Finally, we obtained five modules after WGCNA analysis. Through the pathway enrichment analysis of these modules, we found their association with glycerophospholipid metabolism, arachidonic acid metabolism, carbon metabolism, glycolysis/gluconeogenesis, and pyrimidine/purine metabolism. All these metabolic pathways have a clear relationship with tumors [30–33]. Moreover, key metabolism processes such as glycolysis participate in multiple aspects of breast cancer. For instance, as a core regulator of glycolysis, PDK1 has been found to reprogram stem cells under hypoxia circumstances in breast cancer [34]. Future investigations are still required to elucidate the role of these metabolism pathways in the progression of breast cancer.

Analysis of the correlation between each module and immune infiltration indicated certain relationships between glycerophospholipid metabolism and most of the immune cells. At present, the relationship between glycerophospholipid metabolism and immune cells requires additional in-depth research. However, in our analysis, arachidonic acid metabolism demonstrated a significant correlation with endothelial cells. This conclusion was confirmed by Monika Ermert et al. [35]. In addition, it should be noted that the current analysis is based on transcriptome data. The core or periphery of the tumor may show very different infiltration and immune cell composition. Therefore, this part of the results needs specific follow-up experiments to verify. Next, we also used the GSVA algorithm to assess tumor-related phenotype scores (cell proliferation, apoptosis, and EMT) in breast cancer patients. The final analysis results suggested a significant correlation between glycerophospholipid metabolism and cell proliferation. Glycerophospholipids are key molecules that help cell structures participate in the regulation of many cellular processes. Phospholipid metabolism is the main activity that cells participate in throughout the growth process. In tumor research, many studies have also shown that glycerophospholipids are a marker of tumorigenesis [30, 36, 37].

We further explored the possible regulatory factors of these differential genes. We found that the expression of 17 genes was affected by their mutations. Secondly, a total of 45 genes were negatively regulated by miRNA. Finally, we investigated whether methylation of the gene promoter region affected gene expression. Overall, 90 genes might be regulated by their methylation. Specifically, methylation and miRNA jointly regulated 14 genes while mutation and methylation jointly regulated one gene (PIK3R1). Epigenetic regulations have been suggested as a key regulator of metabolism in breast cancer. For example, knockdown of circDENND4C suppresses glycolysis, migration, and invasion by increasing miR-200b/c under hypoxia in breast cancer cells [38]. It has been suggested that somatic mutations of PIK3R1 (17%), one of the key genes of lipid metabolism, are prevalent and diverse in breast cancer patients [39]. In addition, miR-155 positively modulates glucose metabolism by the PIK3R1/FOXO3a/cMYC pathway in breast cancer [40]. As reversible and plastic regulations, epigenetic alternations are more amenable to therapeutic intervention than more unidirectional genetic alterations [41].

Finally, although in our article, a comprehensive analysis of the role of metabolism-related genes in BRCA has been carried out, the current analysis is mainly based on sequencing data, and any follow-up would need basic experiments for verification. At the same time, based on the difference of race, a study into metabolism-related genes among different races may also have some different effects.

## Conclusion

According to comprehensive analysis of metabolic genes in breast cancer based on multi-omics data of somatic mutation, copy number variation, mRNA expression, miRNA expression, and DNA methylation, we identified a series of differentially expressed metabolic genes. Metabolic genes are associated with tumor-related immune cells and clinical parameters. The potential application of targeting metabolic genes in clinical therapy requires further study to clarify.

## Data Availability

The original contributions presented in the study are included in the article/Supplementary Material, further inquiries can be directed to the corresponding author.

## Supplementary Material

Supplementary Figure S1 The expression difference of metabolism related TMB in pam50 Molecular classification.

Supplementary Figure S2 The expression difference of metabolism related Fraction Genome Alternatƒion in pam50 Molecular classification.

Supplementary Figure S3 Conservative analysis of WGCNA module. In conservative analysis, if Zsummayr is greater than 10, it can be considered that this module is stably expressed.
