# Supplementary figures and images for: Comprehensive Analysis of Metabolic Genes in Breast Cancer Based on Multi-Omics Data
Source: Pathol Oncol Res. 2021 Aug 2;27:1609789. doi: 10.3389/pore.2021.1609789 (PMC8366497; doi:10.3389/pore.2021.1609789)

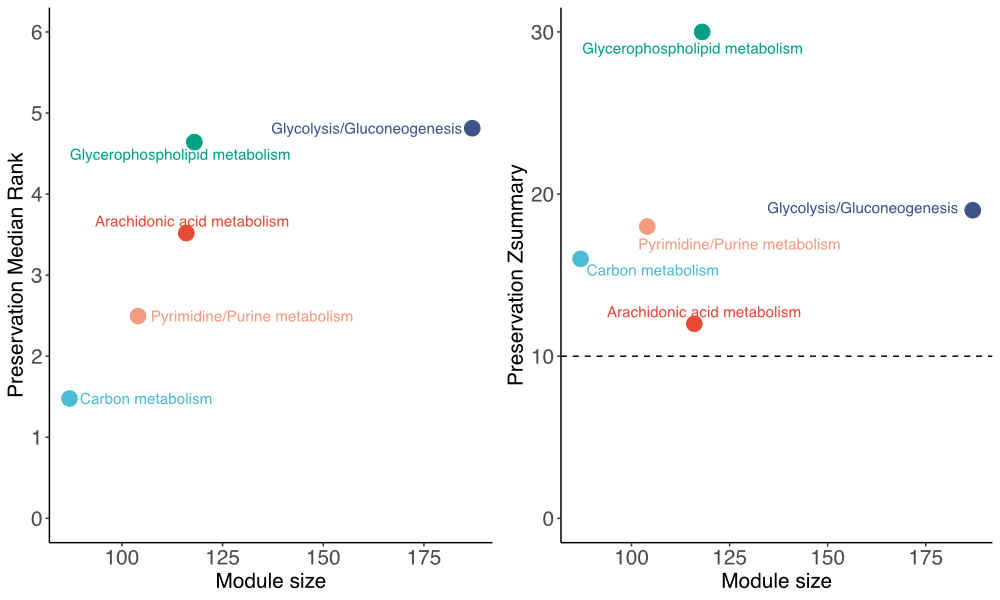

Supplement: Supplementary file 1 [file image3.tiff]

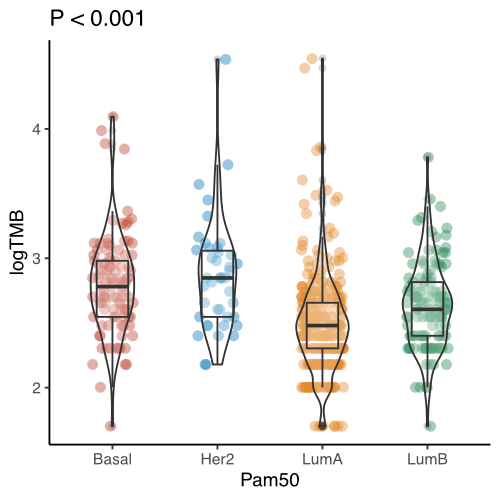

Supplement: Supplementary file 2 [file image1.tiff]

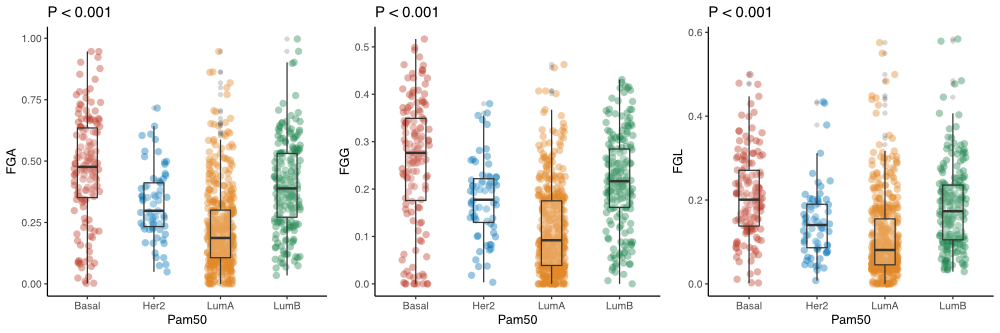

Supplement: Supplementary file 3 [file image2.tiff]
